# Design, Synthesis, and Use of Peptides Derived from Human Papillomavirus L1 Protein for the Modification of Gold Electrode Surfaces by Self-Assembled Monolayers

**DOI:** 10.3390/molecules22111970

**Published:** 2017-11-14

**Authors:** John Alejandro Lara Carrillo, Ricardo Fierro Medina, Juan Manríquez Rocha, Erika Bustos Bustos, Diego Sebastián Insuasty Cepeda, Javier Eduardo García Castañeda, Zuly Jenny Rivera Monroy

**Affiliations:** 1 Department of Pharmacy, Universidad Nacional de Colombia, Carrera 45 No 26-85, Building 450, Office 213, 11321 Bogotá, Colombia; jalaraca@unal.edu.co (J.A.L.C.); jaegarciaca@unal.edu.co (J.E.G.C.); 2 Department of Chemistry, Universidad Nacional de Colombia, Carrera 45 No 26-85, Building 450, Office 334, 11321 Bogotá, Colombia; rfierrom@unal.edu.co (R.F.M.); dsinsuastyc@unal.edu.co (D.S.I.C.); 3 Department of Research, Centro de Investigación y Desarrollo Tecnológico en Electroquímica, S.C., Parque Tecnológico Querétaro, Sanfandila, Pedro Escobedo, 76703 Querétaro, Mexico; jmanriquez@cideteq.mx (J.M.R.); ebustos@cideteq.mx (E.B.B.)

**Keywords:** modified gold electrodes, self-assembled monolayer, synthetic peptides, ferrocene motif

## Abstract

In order to obtain gold electrode surfaces modified with Human Papillomavirus L1 protein (HPV L1)-derived peptides, two sequences, SPINNTKPHEAR and YIK, were chosen. Both have been recognized by means of sera from patients infected with HPV. The molecules, Fc-Ahx-SPINNTKPHEAR, Ac–C–*Ahx*-(Fc)KSPINNTKPHEAR, Ac–C–*Ahx*-SPINNTKPHEAR(Fc)K, C–*Ahx*–SPINNTKPHEAR, and (YIK)_2_–*Ahx*–C, were designed, synthesized, and characterized. Our results suggest that peptides derived from the SPINNTKPHEAR sequence, containing ferrocene and cysteine residues, are not stable and not adequate for electrode surface modification. The surface of polycrystalline gold electrodes was modified with the peptides C-Ahx-SPINNTKPHEAR or (YIK)_2_-Ahx-C through self-assembly. The modified polycrystalline gold electrodes were characterized via infrared spectroscopy and electrochemical measurements. The thermodynamic parameters, surface coverage factor, and medium pH effect were determined for these surfaces. The results indicate that surface modification depends on the peptide sequence (length, amino acid composition, polyvalence, etc.). The influence of antipeptide antibodies on the voltammetric response of the modified electrode was evaluated by comparing results obtained with pre-immune and post-immune serum samples.

## 1. Introduction

According to the WHO (World Health Organization), cervical cancer is the second most common cancer among women worldwide. Every year, more than 270,000 women die from cervical cancer, and more than 85% of these deaths are in low- and middle-income countries [1,2,3,4]. Latin America and the Caribbean region account for 14.6% of the worldwide total of cervical cancer cases. Recurrent infections with specific serotypes of Human Papillomavirus (HPV) (i.e., 16, 18, 45, and 31) are involved in nearly 80% of invasive cervical carcinoma cases [5]. The HPV L1 protein is the most abundant protein and forms the main structure of the icosahedral viral capsid, which is largely responsible for interaction with the cell surface receptor [6,7,8]. Nowadays, immunization with L1 virus-like particles (VLPs) is used as a prophylactic vaccine, which induces the circulation of neutralizing antibodies against HPV 6/11/16/18 serotypes [8]. However, a diagnosis of late-stage cancer combined with the inability to receive treatment condemns many people to unnecessary suffering and early death [9]. The invasive diagnostic tests are poorly accepted by the population, especially in developing countries. Limited access to effective screening means that the disease is often not identified until it is well-advanced and symptoms develop. In addition, the prospects for the treatment of such a late-stage disease may be poor, resulting in a higher rate of death from cervical cancer in these countries [2]. In this context, developing new diagnostic tests (noninvasive, inexpensive, and accessible to the people), could help to develop fast screening and treatment.

The development of biosensors applied to clinical tests has been directed towards the search for high-performance systems with further integration and miniaturization, approaching the molecular level. Biosensors compared with conventional methods have several potential advantages, such as (i) a less traumatic procedure; (ii) increased test speed; (iii) the possibility of miniaturization; (iv) versatility for application in rural regions; and (v) reduced testing costs [10]. The use of biosensors for the diagnosis of HPV infection is a research field that is in an early stage. Recently, it has been demonstrated that the immobilized peptide derived from HPV-16-L1 on a glassy carbon electrode detects antibodies generated against HPV-16. The antigen–antibody complex induces a current drop in the square wave voltammetry signal [11]. Peptides can be used for generating self-assembled monolayers (SAMs) over gold surfaces [12], allowing one to take advantage of specific molecular recognition, such as an antibody–antigen [13,14] with biological and analytical interest, with multiple applications in different fields, such as clinical, industrial, and environmental analysis, among others [15,16]. Peptides can be physically adsorbed (trapped into membranes, electrostatically adsorbed, adsorbed through hydrophobic interactions, etc.) on different electrode surfaces, but it is not an affinity interaction. In this context, we previously designed a bioelectrochemical immunosensor; specifically, the synthetic peptide HPV-1: Fc–Ahx–SPINNTKPHEAR was physically immobilized on a gold electrode, and the coating was done through the drop-casting method, using the Nafion polymer. HPV-1 was designed containing a ferrocene probe, a spacer, and a sequence derived from L1-HPV protein [17]. The electrochemical behavior of the modified electrode was examined via cyclic voltammetry in order to evaluate the redox behavior of the ferrocene moiety. Changes in such behavior upon addition of the serum samples suggested that the fabricated bioelectrochemical sensor was able to recognize the interaction between antipeptide antibodies and the immobilized HPV-1 peptide with high selectivity and sensitivity.

It is well-known that peptides interact with various metals, such as gold, silver, platinum, or copper oxide surfaces, according to the affinity of certain amino acids for these surfaces. Specifically, thiol-containing peptides (Cys residue) bind to metal surfaces, particularly gold. Depending on the solvent composition and/or pH, carboxylates or amino groups may also interact with the surface, which could involve conformational changes in the adsorbed peptide. The conformational change will depend on the morphology of the surface on which the Self-Assembled Monolayers (SAM) is formed [18,19]. SAMs using peptides have several advantages, including; (i) easy synthesis; (ii) low cost; (iii) design flexibility (desired functional groups can easily be incorporated on the surface); (iv) quite stable binding due to the strong thiol-gold interaction; (v) low number of molecules required for the monolayer formation; and (vi) reasonable stability of the monolayer [20,21,22,23,24].

In the present paper, we report the design, synthesis and electrochemical study of self-assembled monolayers of two synthetic peptides derived from the HPV-L1 protein whose original sequences are specifically recognized by antibodies against HPV-16 [25,26]. These peptides, HPV-3 and HPV-4, have one cysteine residue at the C-terminal or N-terminal end, respectively. Their side-chain thiol group is responsible for the peptide chemisorption over polycrystalline gold surfaces [16,27]. Peptides self-assembled over gold electrodes were characterized via Fourier transform infrared (FT-IR) spectroscopy and electrochemical techniques, which allowed us to evaluate the thermodynamic parameters of the electrode, surface coverage factor, and chemical stability.

## 2. Results

### 2.1. Design and Synthesis of HPV-Derived Peptides

Our approach was to couple antigenic peptides derived from HPV proteins to an electrode surface. We selected two antigenic sequences that were previously reported, (i) the peptide SPINNTKPHEAR, which was recognized by antibodies from patients infected with HPV, who already have lesions on the cervix [26], and (ii) the sequence YIK, which was identified as the sequence to which the monoclonal antibodies 8C4, 3D1, 1D6, and 5A4 bind. These monoclonal antibodies recognize viral antigens and HPV-16 L1 protein [25]. Table 1 shows the peptides used in this research.

Based in our previous results [17], we designed two peptides: HPV-2 and HPV-3. Both of them contain: (i) an antigenic sequence (underlined); (ii) a cysteine residue, which was introduced since it is well-known that compounds containing thiol groups are strongly coordinated with a variety of metals, including gold, silver, platinum, and copper [28,29]. The structure of a self-assembled monolayer mainly depends on the morphology of the metal. Au is widely used for the formation of monolayers. The cysteine residue was introduced at the N or C end of the antigenic sequence; (iii) a spacer (aminohexanoic residue, *Ahx*), which separates the Cys residue from the antigenic sequence; and finally; (iv) a lysine residue, which allows attaching the ferrocene (Fc) motif (electrochemical probe) to its epsilon amine group. The designed peptides were obtained via solid-phase peptide synthesis, purified via solid-phase extraction, and characterized via reverse phase high performance liquid chromatography (RP-HPLC), electrospray ionization mass spectrometry (ESI-MS) or matrix-assisted laser desorption/ionization-time of flight mass spectrometry (MALDI-TOF MS).

**Figure 1:**
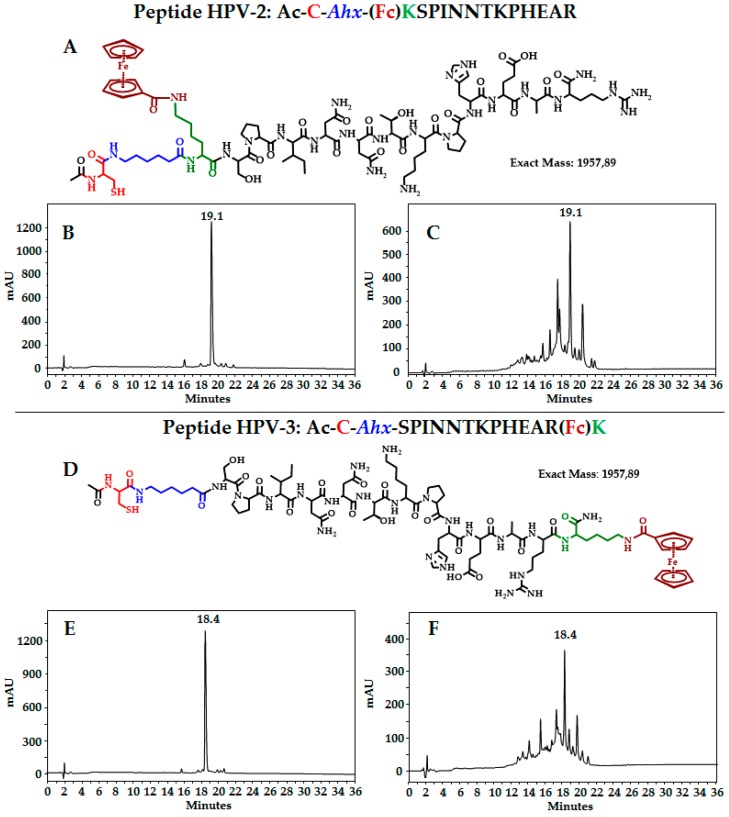
Peptides derived from Human Papillomavirus L1 protein (HPV L1). Both sequences contain (i) a cysteine residue (in red); (ii) an aminohexanoic residue (in blue); (iii) lysine residue (in green); and (iv) the ferrocene motif (in brown). Structure of peptide HPV-2: (**A**) reverse phase high performance liquid chromatography (RP-HPLC) profile of the purified product (**B**) before, and (**C**) after the lyophilization process. Structure of peptide HPV-3: (**D**) RP-HPLC profile of the purified product (**E**) before and (**F**) after the lyophilization process.

**Figure 2:**
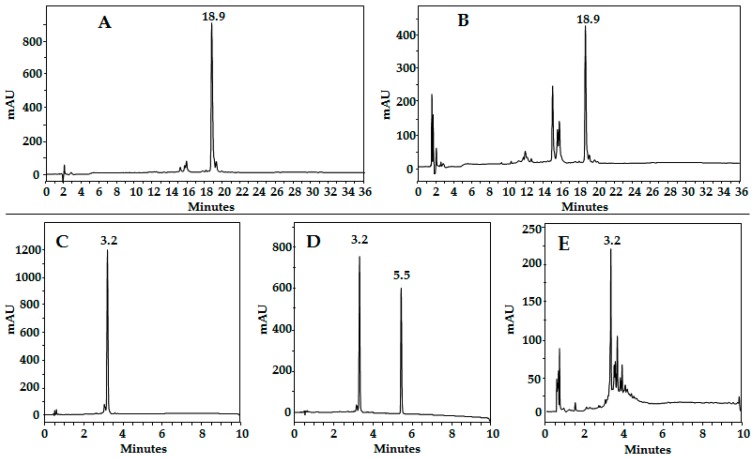
Stability of peptides derived from SPINNTKPHEAR. Chromatographic profile of peptide HPV-1 ((**A**), *t*_R_: 18.9 min); (**B**) HPV-1 mixed with cysteine solution and lyofilized; (**C**) peptide HPV-5 C–Ahx–SPINNTKPHEAR; (**D**) HPV-5 (*t*_R_: 3.2 min) mixed with free Ferrocenecarboxylic acid (*t*_R_: 5.5 min), reaction time 0 h and (**E**) 24 h.

Figure 1 shows the structure of peptide HPV-2 (A) and the chromatographic profile of the purified product (B). ESI MS shows signals at *m*/*z* 981.00 and 654.85, corresponding to [M + 2]^2+^ and [M + 3]^3+^, respectively. After the lyophilization process of the purified peptide HPV-2, the chromatographic profile changes, showing several peaks (C). Similar behavior was observed for peptide HPV-3 (Figure 1D–F). This result suggests that these peptides are not stable. Peptide HPV-1 (0.5 mM) was mixed with cysteine (0.5 mM) (1:1 *v*/*v*), and then the mixture was lyophilized. Interestingly, the chromatographic profile shows undesired species, suggesting HPV-1 degradation (Figure 2A,B). It was also established that peptide HPV-4 mixed with ferrocenecarboxylic acid degraded after 24 h (Figure 2C,D). These results suggest that peptides derived from the SPINNTKPHEAR sequence containing both ferrocene and cysteine residues are not stable.

**Figure 3:**
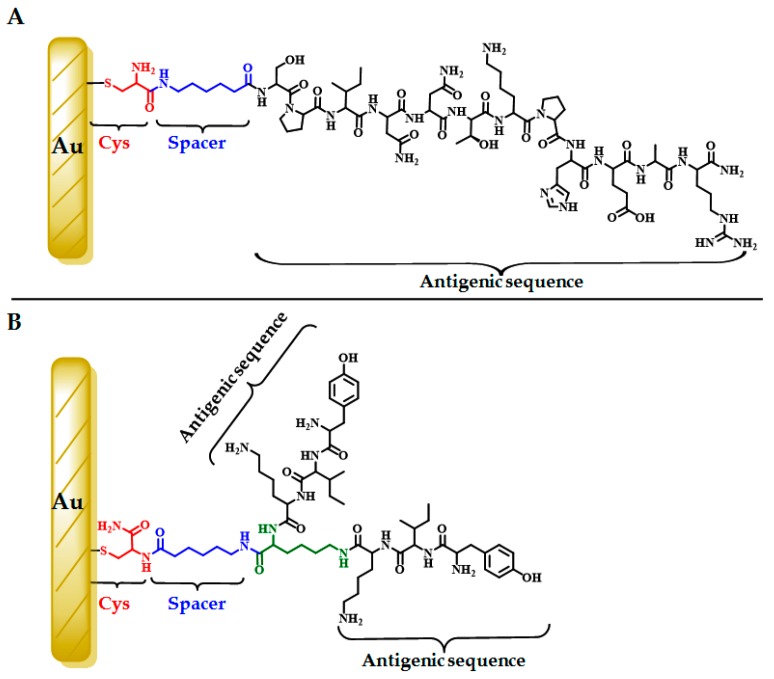
Schematic drawings of the polycrystalline gold electrode modified with (**A**) peptide HPV-4; and (**B**) dimeric peptide HPV-5.

Once it was evident that peptides HPV-2 and HPV-3 have poor stability, it was decided to modify the gold electrodes using the peptides HPV-4 and HPV-5. It should be pointed out that peptide HPV-5 corresponds to a dimer of the YIK sequence. A schematic drawing of the gold electrode modified with the designed peptides is shown in Figure 3.

Designed HPV-4 and HPV-5 peptides were synthesized, purified, and characterized. The chromatographic profiles of purified HPV-4 and HPV-5 peptides showed, in both cases, a main specie at retention time (t_R_) of 3.2 and 3.8 min, respectively. MALDI-TOF mass spectrometry peptides HPV-4 *m*/*z* = 1579.94 (calculated [M + H]^+^: 1578.81) and HPV-5 *m*/*z* = 1171.09 (calculated [M + H]^+^: 1170.70), Figure S1 (Supplementary Materials).

### 2.2. Functionalization of Gold Electrodes Using Peptides HPV-4 and HPV-5

**Figure 4:**
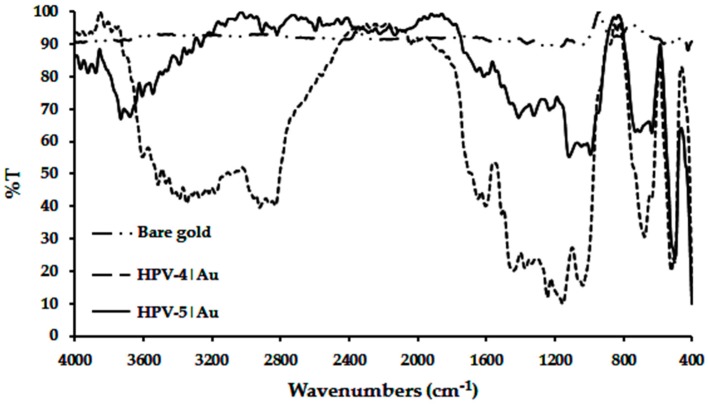
FT-IR spectrum of HPV-4|Au, HPV-5|Au and bare Au electrodes at an incident ray angle of 60°.

Polished polycrystalline gold electrodes were treated with HPV-4 or HPV-5 solution. After the spontaneous self-assembly reaction occurred, the excess of peptides was removed by rinsing, and modified electrodes were characterized via FT-IR using the external reflectance technique with 10°, 20°, 30°, 40°, 50°, 60°, 70°, 80° and 90° as incident ray angles. The FT-IR spectra at 60° shows bands associated with the amide group and the amino acid side-chain functional groups, indicating that the peptide self-assembled over the gold electrodes (Figure 4). Bands in the 3500–3100 cm^−1^ region correspond to N–H stretching in secondary amides. The stretching frequency for the C=O group exhibited in amide I appears in the range of 1630–1510 cm^−1^ [30]. It is important to point out that amide I and amide II bands have been the most frequently used in the confirmation of peptide binding to the electrode [31]. For peptide HPV-5, these bands appear at the incident ray angle of 90°. The bands in the range 1560–1510 cm^−1^ are assigned to N–H bending and N–C=O symmetrical stretching in amide II. For the surfaces analyzed, these bands appear at the incident ray angle of 10°. For peptide HPV-4, the spectra at the lower incident angles (10° and 20°) do not exhibit bands in the region 4000–1660 cm^−1^, and at the large incident angles (80° and 90°), the noise is greater. This could be attributed to the inclination of the peptide molecules when they have been organized on a gold surface. This makes it possible to determine the principal bands of peptides and their side chains at intermediate incident angles, which possibly demonstrates that HPV-5 covered a greater area of the gold than HPV-4. The bending vibrations of N–H and C–N stretching in amide III appear at 1244 and 1232 cm^−1^, respectively [31,32]. The asymmetric stretching frequencies for the -CH_2_- groups that are present in the 6-aminohexanoic residue side chain and the backbone peptide appear in the region of 2922–2833 cm^−1^. The C–N stretching band from primary, secondary and tertiary aliphatic amines is shown at 1037 cm^−1^ in the FT-IR spectra for the peptide HPV-4. The (COO-)_sy_ and ν(C-C) stretching, with assigned bands 1411 and 992 cm^−1^, are in the carboxylate group and the aliphatic chain, respectively, for peptide HPV-5. Finally, the presence the C–S stretching band in the region 630–730 cm^−1^ and the absence of an S–H stretching frequency at 2550–2600 cm^−1^ indicate that peptide molecules were self-assembled on the gold surface via the formation of gold–sulfur linkages. Assignments are tabulated in Table S1 (Supplementary Materials).

**Figure 5:**
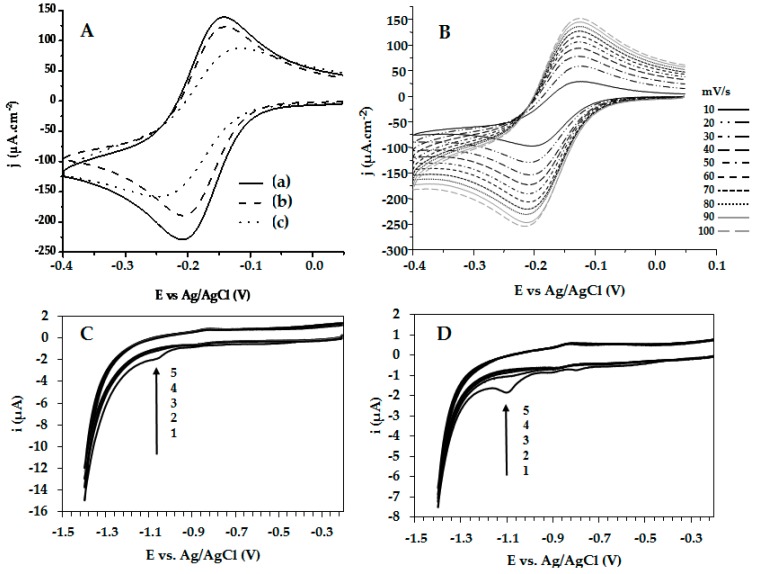
Cyclic voltammograms of the gold electrodes using a scan rate of 80 mV/s (**A**): bare Au (curve a), HPV-4|Au (curve b) and HPV-5|Au (curve c). Cyclic voltammograms of HPV-4|Au electrode at different scan rate (**B**). All measurements were performed in 1 mM Ru(NH_3_)_6_Cl_3_ in 0.50 M KCl at RT. Electrochemical desorption of HPV-4 (**C**) and HPV-5 (**D**) using 1, 2, 3, 4, 5, and 6 cyclic voltammograms in the presence of potassium hydroxide (0.5 M) at 20 mV/s.

The electrodes were also electrochemically characterized before and after modification by cyclic voltammetry. The conventional three-electrode system was used to determine the effect of working electrode surface modification. The electrochemical behavior observed in a solution of 1 mM $\mathrm{Ru}{\left({\mathrm{NH}}_{3}\right)}_{6}{\mathrm{Cl}}_{3}$ (probe molecule, redox pair $\mathrm{Ru}{\left({\mathrm{NH}}_{3}\right)}_{6}^{3+}/\mathrm{Ru}{\left({\mathrm{NH}}_{3}\right)}_{6}^{2+}$) in 0.5 M potassium chloride (KCl) as a supporting electrolyte was recorded at 80 mV/s. Measurements were made between the potential at zero current and −0.4 V. For the bare electrode (Figure 5A, curve a), a typical reversible cyclic voltammogram was found. The active area (A_act_) for the bare electrode was 0.0240 cm^2^; the real surface area (A_real_) was calculated from the charge required to form an AuO monolayer, and it corresponded to 0.0257 cm^2^ (Supplementary Materials, Figure S2). The relationship between A_real_ and A_act_, corresponding to the roughness factor, was 1.07. This value indicates that the bare Au electrode was planar, without roughness.

The cyclic voltammetry of the modified electrodes showed cathodic and anodic current densities lower than those exhibited by the bare electrode (Figure 5A, curves b and c), produced by the presence of HPV-4 and HPV-5 peptides, respectively, on the electrode surface. For the probe system used, the HPV-5-modified electrode exhibited an irreversible behavior (Δ*E* = |E_ap_ − E_cp_| = 130 mV and |i_ap_/i_cp_| = 1.8), while the HPV-4-modified electrode exhibited a ΔE of 69 mV and |i_ap_/i_cp_| = 1.6, this being closer to that corresponding to a reversible process. E_cp_ and E_ap_ are independent of the scan rate (Figure 5B); therefore, system can be considered as reversible [28]. Thermodynamic parameters obtained from the voltammograms (Figure 5A) are shown in Table 2.

Adsorbed thiols on gold surfaces are reductively desorbed in a basic medium. The desorption potential is dependent on both the sulfur–gold bond strength and the molecular interactions between adsorbed species. The desorption process can be described by: $AuRS+e^{-}⇆Au+RS^{-}$ (redox reaction) [28,29,30]. The reductive electrochemical desorption of the self-assembled peptides (HPV-4 and HPV-5) was obtained via cyclic voltammetry in 0.5 M potassium hydroxide (KOH), using a scan ranging from null potential current (E_i_ = 0) to −1.4 V vs. Ag/AgCl. In the first cycle, for both modifications, a reductive desorption wave was observed. For the HPV-4 peptide, the reduction potential was −1.06 V vs. Ag/AgCl, and for the HPV-5 peptide it was −1.10 V vs. Ag/AgCl. These waves disappeared in the second cycle, indicating that the peptides were adsorbed on the gold surface through the covalent bond Au-S (Figure 5C,D). In the case of desorption of the HPV-5 peptide from the Au electrode (Figure 5D), two additional electrochemical signals, at −0.9 and −0.8 V vs. Ag/AgCl, can be seen, which indicates three different molecular configurations over the gold electrode. This can be attributed to the interaction of free amine groups of the sequence with the gold surface, as is shown in Figure 3B.

The surface coverage associated with each peptide was calculated using Γ = Q/nFA, where Q is the charge for reductive desorption (0.29 and 1.2 µC for HPV-4 and HPV-5, respectively), n is 1 mol of $e^{-}$, F is the Faraday constant (96.485 C/mol), and A is the real surface area [29]. As Table 2 shows, the surface coverage of the HPV-4 peptide ($9.6\times 10^{-11}$ mol/cm^2^) was lower than the one established for the HPV-5 peptide ($40\times 10^{-11}$ mol/cm^2^). This could be caused by steric hindrance, since the HPV-4 is bulkier than the HPV-5 peptide, obstructing the adsorption and organization of more peptide molecules over the surface (Figure 3). In contrast, the current density was less for the HPV-5|Au than for the HPV-4|Au electrode, indicating less available area for the electronic transfer process for the HPV-5|Au electrode, caused by the high surface coverage (Table 2).

The pH effect (in the supporting electrolyte) on electrochemical measurements using HPV-4|Au and HPV-5|Au electrodes was studied (Supplementary Materials, Figure S3). Two probe molecules were tested: (i) $\mathrm{Ru}{\left({\mathrm{NH}}_{3}\right)}_{6}^{3+}$ (positively-charged complex) and (ii) ferrocene (neutral). At pH 2–5, a reduction in the current density over the modified electrode was observed for the $\mathrm{Ru}{\left({\mathrm{NH}}_{3}\right)}_{6}^{3+}$ probe (Figure S2A). Peptides assembled over gold electrodes contain groups that can be ionized depending on the pH values (Figure 3); i.e., for both peptides, at low pH values a positive net charge is generated, which is caused by the Lys and Arg protonation, His side chains, and the N-terminal amine group. This positive charge could repel the $\mathrm{Ru}{\left({\mathrm{NH}}_{3}\right)}_{6}^{3+}$ complex, causing the reduction in current density. For a neutral probe molecule such as ferrocene, the current density is low and constant over all the pH range studied, measured with the bare electrode and both modified electrodes (Figure S2B).

**Figure 6:**
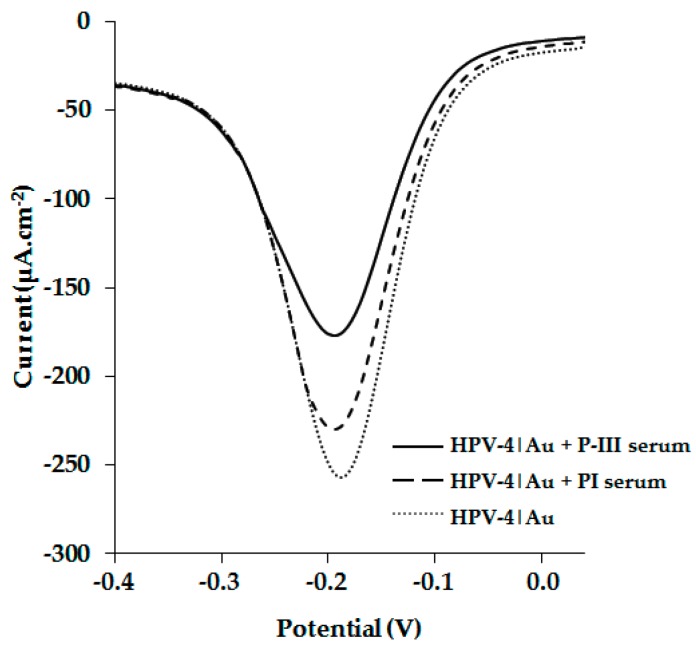
Polyclonal anti-HPV-4 detection using HPV-4|Au electrode by square-wave voltammetry (SWV). For unmodified electrode the minimal current density was −575 µA.cm^−2^ (at ~0.2 V). All measurements were performed in 1 mM Ru(NH_3_)_6_Cl_3_ in 0.50 M KCl at RT.

New Zealand rabbits were immunized using a polymeric peptide, CGSPINNTKPHEARGC, and rabbit sera were evaluated by ELISA; the post III serum specifically recognized peptide HPV-4 with a 1:25,600 dilution. Through the use of the square-wave voltammetry (SWV) technique, we compared the behavior of the HPV-4|Au electrode in the presence of preimmune (PI) or post-III (PIII) sera. The results showed that it was possible to observe the antigen–antibody interaction (Figure 6). The antigen–antibody complex formation induces a significant current drop compared with the HPV-4|Au electrode, in the presence or absence of PI serum. Our results suggest that HPV-4|Au and HPV-5|Au surfaces could be useful to develop electrochemical detectors for recognizing antibodies from sera of patients with HPV infection.

## 3. Materials and Methods

### 3.1. Reagents and Materials

The gold electrodes were purchase from DropSens (Asturias, Spain) and BASi (West Lafayette, IN, USA), Rink amide resin (0.46 meq/g), Fmoc-Arg(Pbf)-OH, Fmoc-Lys(Boc)-OH, Fmoc-Glu(OtBu)-OH, Fmoc-Ala-OH, Fmoc-Ile-OH, Fmoc-Thr(tBu)-OH, Fmoc-Ser(tBu)-OH, Fmoc-Asn(Trt)-OH, Fmoc-Pro-OH, Fmoc-Cys(Trt)-OH, Fmoc-His(Trt)-OH, Fmoc-Lys(ivDde)-OH, Fmoc-Lys(Fmoc)-OH, 6-Fmoc-aminohexanoic acid (Fmoc-Ahx-OH), Fmoc-Tyr(tBu)-OH, 1-hydroxybenzotriazole (HOBt), and *N,N*-dicyclohexylcarbodiimide (DCC) were purchased from AAPPTec (Louisville, KY, USA). *N,N*-diisopropylethylamine (DIPEA), triisopropylsilane (TIPS), 1,2-ethanedithiol (EDT), acetic anhydride, piperidine, pyridine, ninhydrin, phenol, ferrocene, Ferrocenecarboxylic acid, Freund’s incomplete and complete adjuvant, and KCN were purchased from Sigma-Aldrich (St. Louis, MO, USA). Methanol, diethyl ether, *N*,*N*-dimethylformamide (DMF), absolute ethanol, dichlorometane (DCM), acetonitrile (AcN), isopropyl alcohol (IPA), and trifluoroacetic acid (TFA) were obtained from Honeywell-Burdick & Jackson (Muskegon, MI, USA). Analytical grade KOH, KCl, Ru(NH_3_)_6_Cl_3_, K_3_Fe(CN)_6_, and H_2_SO_4_ were acquired from J.T. Baker. The pH was adjusted with either KOH or HCl solutions obtained from J. T. Baker. All aqueous solutions were prepared with deionized water (ρ ≥ 18 MΩ cm^−1^). All reagents were used without further purification.

### 3.2. Peptide Synthesis and Characterization

Peptides were synthesized using the Fmoc/tBu methodology [33,34,35]. Briefly, Rink amide resin was used as a solid support. (i) The Fmoc group removal was carried out by treatment with 20% 4-methylpiperidine in *N,N* dimethylformamide (DMF) at room temperature (RT) for 10 min twice. Removal of the ivDde group was performed using 2% hydrazine in DMF for 3 min three times; (ii) For the coupling reaction, 0.21 mmol of Fmoc-amino acid was preactivated with *N,N*-dicyclohexylcarbodiimide (DCC)/1-hydroxybenzotriazole (HOBt) (0.20/0.21 mmol) in DMF. The preactivation mixture was shaken at RT for 15 min. After that, the activated Fmoc-amino acid was added to a reactor containing deprotected resin; the coupling reaction was shaken for two hours at RT, and then the resin was washed with DMF and DCM. Fmoc group elimination and the incorporation of each amino acid were confirmed through the ninhydrin test; (iii) Side chain deprotection reactions and peptide separation from the solid support were carried out with a cleavage cocktail containing TFA/water/TIPS/EDT (93%/2%/2.5%/2.5% *v*/*v*/*v*/*v*), the reaction was shaken for 6 h at RT, and then mixture was filtered and the solution was collected. Crude peptides were precipitated by treatment with cool ethyl ether; (iv) The crude products were purified via solid-phase extraction (SPE), and the purified products were analyzed using RP-HPLC and MALDI-TOF MS, as described in Supplementary Materials (Analytical Methods).

### 3.3. Electrode Modification and Characterization by FT-IR

Polycrystalline gold electrodes were polished to a mirror-like finish with 1.0, 0.3, and 0.05 µm alumina slurry on pads (Buehler, Binghamton, NY, USA). The alumina excess was removed by rinsing and sonication of the electrode in deionized water for 10 min. The electrochemical cleaning was performed for 20 cycles between 0 and +1.65 V relative to the Ag/AgCl electrode in 0.50 M H_2_SO_4_ solution at a 40 mV·s^−1^ scan rate [28].

Peptide self-assembly was generated by immersion of bare gold electrodes in 1 mM peptide solution in methanol for 18 h at RT, as described by Ulman [29]. Then the electrodes were carefully washed with methanol and deionized water in order to remove physically adsorbed molecules. Fourier transform infrared (FT-IR) measurements were performed in order to characterize the self-assembled peptide on a screen-printed gold electrode using a Thermo Nicolet spectrometer (150 scans, energy scanning from 400 to 4000 cm^−1^) and OMNIC software version 6.1a on a Pentium-based PC. All measurements were performed at RT.

### 3.4. Electrochemical Measurements

Electrochemical measurements using cyclic voltammetry (CV) were carried out with a BASi electrochemical analyzer (BASi Corporate Headquarters, West Lafayette, IN, USA) and a conventional three-electrode system comprising a reference electrode of Ag/AgCl 3.0 M NaCl, a bare or modified working electrode, and a platinum wire as the auxiliary electrode. CV experiments were performed in 1 mM Ru(NH_3_)_6_Cl_3_ in 0.50 M KCl at RT. Peptide desorptions were performed using six cycles of CV in the presence of KOH (0.5 M) at 20 mV/s. SWV was conducted in 1 mM Ru(NH_3_)_6_Cl_3_ in 0.50 M KCl (supporting electrolyte) at RT. The pulse amplitude of SWV was set at 25 mV and potential step 4 mV; potential was scanned from 50 mV to 400 mV. The frequency of SWV was 15 Hz.

### 3.5. Rabbit Immunization

New Zealand strain rabbits were injected on days 0, 20, and 40 with 500 µg of CGSPINNTKPHEARGC polymerized peptide mixed with Freund’s incomplete adjuvant. Bleeding was carried out on days 0 (PI), 60 (P-II), and 90 (P-III), and sera were collected. Immunizations and bleeding were performed in accordance with the Colombian Ministry of Public Health handing procedures for animals [36].

### 3.6. Enzyme-Linked Immunosorbent Assay (ELISA)

Ninety-six-well polysorb plates were covered with peptides CGSPINNTKPHEARGC or HPV-4 (100 μL, 10 μg/mL) overnight at 4 °C and then incubated at 37 °C for one hour. Then the dishes were treated with 200 μL of Solution A (5% skimmed milk, 0.5% Tween 20 in PBS), for one hour at 37 °C. Then, 100 μL (1:100 dilution) of rabbit sera (PI or P-III) prepared in Solution A were added, and the dishes were incubated for one hour at 37 °C. dishes were washed and Following they were incubated with 100 μL of peroxidase-coupled anti-rabbit IgG secondary antibody (1:5.000) diluted in Solution A, for one hour at 37 °C; a peroxidase substrate solution was added in order to reveal the reaction, according to the manufacturer’s recommendations. Optical density (OD) was measured at 620 nm.

## Figures and Tables

**Table 1:** Designed and synthesized Peptides.

Original Peptide	Derived Sequence	Peptide Code
SPINNTKPHEAR	Fc–*Ahx*–SPINNTKPHEAR	HPV-1
	Ac–C–*Ahx*–(Fc)KSPINNTKPHEAR	HPV-2
	Ac–C–*Ahx*–SPINNTKPHEAR(Fc)K	HPV-3
	C–*Ahx*–SPINNTKPHEAR	HPV-4
YIK	(YIK)_2_–*Ahx*–C	HPV-5

**Table 2:** Thermodynamic parameters for the process $\mathrm{Ru}{\left({\mathrm{NH}}_{3}\right)}_{6}^{3+/2+}$ using for the current measuring bare Au, HPV-4|Au and HPV-5|Au electrodes.

Electrode	Г (mol cm^−2^)	i_cp_ (µA)	i_ap_ (µA)	i_cp_/i_ap_	E_cp_ (mV)	E_ap_ (mV)	ΔE (mV)
Au-bare	0	−5.145	4.212	1.2	−205	−141	64
HPV-4|Au	$9.6\times 10^{-11}$	−4.567	2.975	1.6	−208	−139	69
HPV-5|Au	$40\times 10^{-11}$	−3.803	2.108	1.8	−247	−117	130

## Supplementary Materials

Supplementary materials are available online.
