# Supplementary material for: Design, Synthesis, and Use of Peptides Derived from Human Papillomavirus L1 Protein for the Modification of Gold Electrode Surfaces by Self-Assembled Monolayers
Source: Molecules. 2017 Nov 14;22(11):1970. doi: 10.3390/molecules22111970 (PMC6150238; doi:10.3390/molecules22111970)
Supplement: Supplementary file 1 [file molecules-22-01970-s001.pdf]

molecules

Supplementary Materials

Design, Synthesis, and Use of Peptides Derived from Human Papillomavirus L1 Protein for the Modification of Gold Electrode Surfaces by Self-Assembled Monolayers

John Alejandro Lara Carrillo[1], Ricardo Fierro Medina[2], Juan Manríquez Rocha[3], Erika Bustos Bustos[3], Diego Sebastián Insuasty Cepeda[2], Javier Eduardo García Castañeda[1], Zuly Jenny Rivera Monroy[2*]

Correspondence: zjriveram@unal.edu.co; Tel.: +57-1-3165000 (ext. 14436)

Table of contents

Table S1. FT-IR detailed band assignment for Au electrodes modified with **HPV-4** and **HPV-5**.

Figure S1. Characterization of synthetic peptides derived from L1-HPV.

Figure S2. Cyclic voltammogram of the bare gold electrode

Figure S3. Effect of pH on current density.

Calculation of Surface Roughness

Analytical Methods

Table S1. FT-IR detailed band assignment for Au electrodes modified with **HPV-4** and **HPV-5**.

	Self-assembled Peptide	
**Assignment**	**HPV-4**	**HPV-5**
Amide A-B ν(N-H)	3392-3347	3392-3265
Amide I ν(C=O)	1648	1646
Amide II δ(N-H), ν(N-C=O)$_{sy}$	1540	1539
Amide III ν(C-N), δ(N-H)	1244	1232
δ(NH_3^+)$_{asy}$	2043	2222-2134
ν(C-H), alkyl chain (C-H, -CH_2-)	2922-2832	2822
Tyrosine ν(O-H)	----------	3732-3680
Tyrosine τ(O-H)	----------	635
Tyrosine benzene ring vibrations	----------	1614
Threonine ν(O-H)	3606	----------
Histidine ν(N-H)$_{heterocycle}$	3272	----------
Asparagine ν(C=O)	1604	----------
Glutamic acid ν(COO^-)$_{SIM}$	1442	----------
Isoleucine δ(CH_3)$_{SIM}$	1373	----------
Isoleucine ω or τ(CH_3)	----------	1323
Serine δ(O-H)	1244	----------
Lysine ϱ(NH_3^+)	1155	1116
Lysine τ(NH_3^+)	521	500

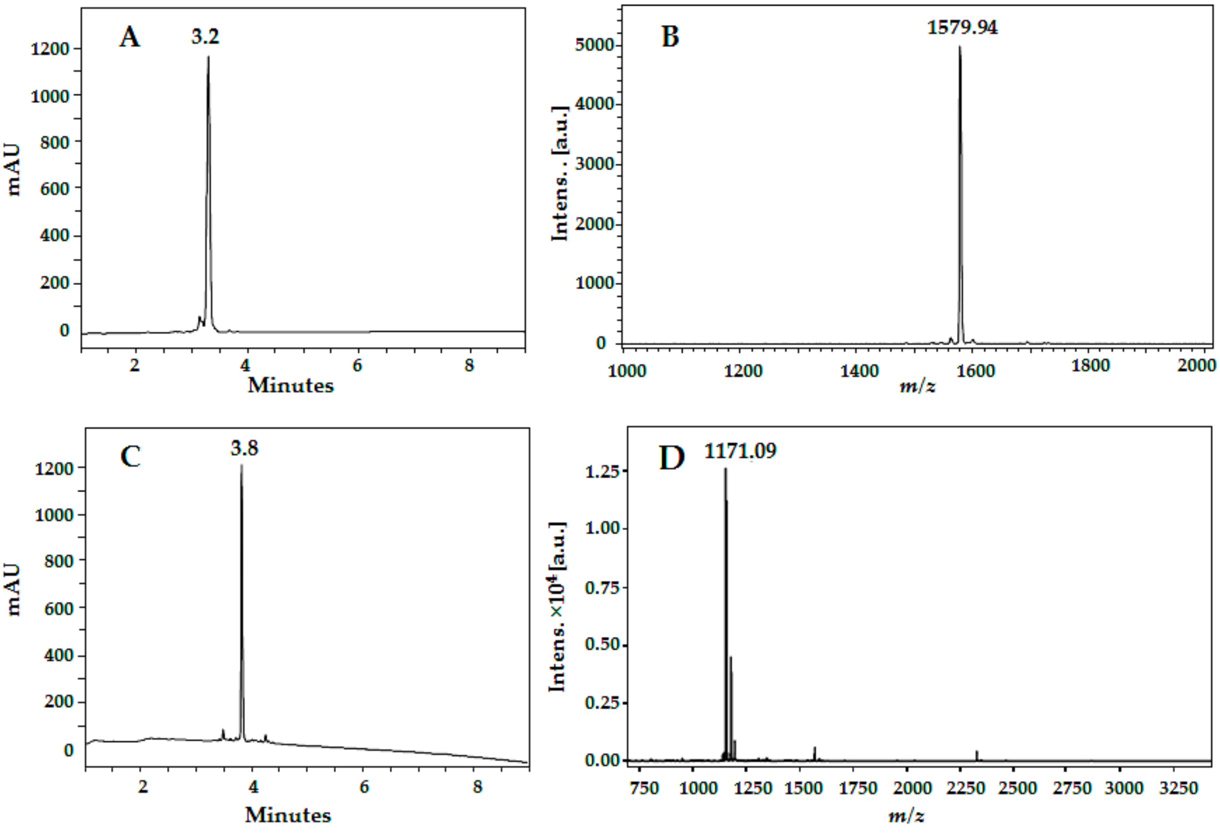

Figure S1. Characterization of synthetic peptides derived from L1-HPV. Peptide **HPV-4**: (A) RP-HPLC profile, (B) MALDI-TOF spectrum. Dimeric peptide **HPV-5**: (C) RP-HPLC profile, (D) MALDI-TOF spectrum.

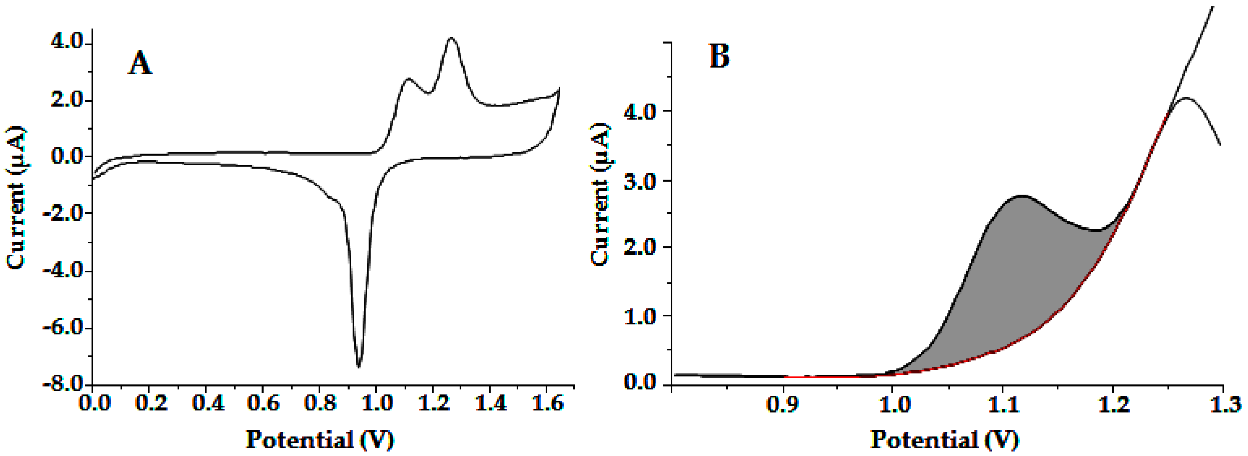

Figure S2. Cyclic voltammogram of the bare gold electrode (A). The electrochemical cleaning was performed for 20 cycles between 0 to +1.65 V relative to the Ag/AgCl electrode in 0.50 M H_2SO_4. For integrating the area under the curve, it was used the enlargement shown in (B).

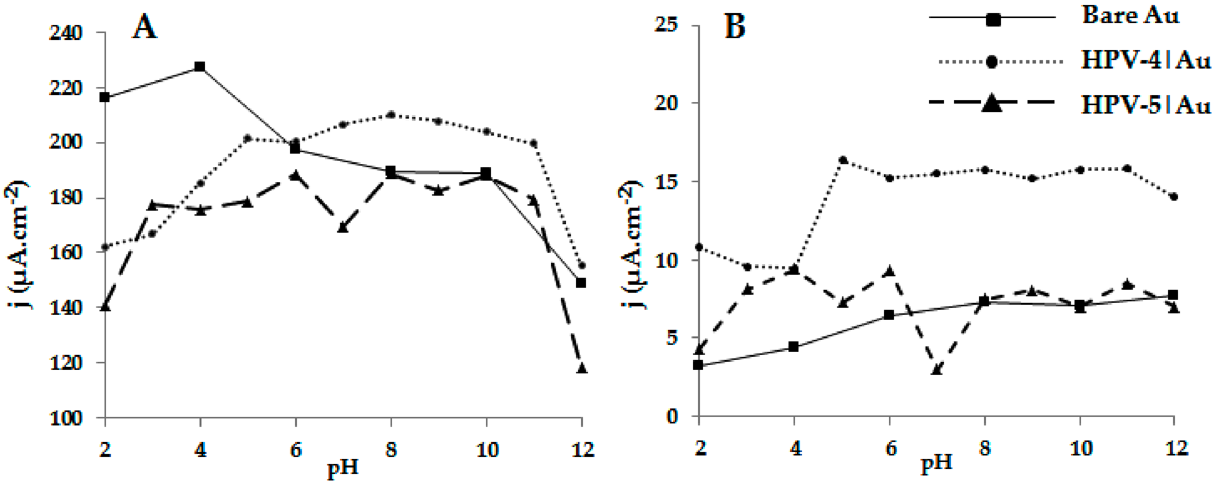

Figure S3. Effect of pH on current density. Measurement were performed at RT using the **HPV-4**|Au (black circles), **HPV-5**|Au (black triangles) and bare Au (black square) electrodes. $Ru(NH_3)_6^{3+}$ (A) and Ferrocene (B) were used as probe molecules.

Calculation of Surface Roughness

Areas were estimated using the Randles–Sevcik equation: $i_p = 2.69 \times 10^5 n^{3/2} D A^{1/2} C v^{1/2}$. Where i_p is the peak current, n corresponds to the number of electrons involve in the reaction, C is the concentration of redox probe in bulk, D is the diffusion coefficient of redox probe, $v^{1/2}$ is the square root of potential scan rate and A is the electroactive area (A_{act}). The calculation was estimated in a scan rate range between 10 and 100mV/s. For real area (A_{real}) determination was used the following equation: $A_{real} = Q_{AuO}(\mu C)/200(\mu C/cm^2)$ the charge associated to develop of a one AuO monolayer when the electrode is oxidized at potentials >0.9 V in sulfuric acid corresponds to 200µC/cm^2. For this reason, it is possible to determine the A_{real}, integrating the area under the curve (Figure S2B) for the formation of the first gold oxide. Surface roughness was calculated as A_{real}/A_{act}.

Analytical Methods

Reverse Phase HPLC.

RP-HPLC analysis was performed on an Agilent 1200 liquid chromatograph (Omaha, Nebraska, USA) with UV-Vis detector (210 nm). For data presented in Figure 2 C-D and Figure 1S, a Merck Chromolith® C18 (50 × 4.6 mm) column was used with a linear gradient applied from 5% to 70% Solvent B (0.05% TFA in ACN) in Solvent A (0.05% TFA in water) for 11.5 min at a flow rate of 2.0 mL/min at room temperature. For data presented in Figure 1 and Figure 2 A,B an Eclipse XDB-C18 column (3.5 µm; 4.6 × 150 mm) was used, using a linear gradient from 5 to 70% Solvent B in Solvent A, and gradient time was 45 min, a flow rate of 1.0 mL/min. In all analysis, 10 µL of sample solution (1 mg/mL), were used.

Peptide Purification.

Molecules were purified using solid-phase extraction columns (SUPELCO LC-18 with 2.0 g resin). SPE columns were activated prior to use with methanol and acetonitrile (containing 0.1% TFA) and equilibrated with water (containing 0.1%TFA). Crude peptides (40mg) were passed through the column, and a gradient was used for their elution. Collected fractions were analyzed using RP-HPLC (as describe above). Fractions that contained pure products were lyophilized.

MALDI-TOF MS.

This analysis was performed on an Ultraflex III TOF-TOF mass spectrometer (Bruker Daltonics, Bremen, Germany) in reflectron mode. 2 µL of peptide solution (1 mg/mL) was mixed with 18 µL of matrix solution (10 mg/mL), and then the mixture (1 µL) was seeded in an MTP384 polished steel target (BrukerDaltonics) and dried. 2,5-dihydroxybenzoic acid or sinapinic acid was used as a matrix, with laser: 500 shots and 25–30% power.
